# Diagnostics and Treatment of Extrameningeal Solitary Fibrous Tumors

**DOI:** 10.3390/cancers15245854

**Published:** 2023-12-15

**Authors:** Anna Maria Janik, Anna Terlecka, Mateusz J. Spałek, Kjetil Boye, Bartłomiej Szostakowski, Paulina Chmiel, Anna Szumera-Ciećkiewicz, Klaudia Bobak, Tomasz Świtaj, Piotr Rutkowski, Anna M. Czarnecka

**Affiliations:** 1 Department of Soft Tissue/Bone Sarcoma and Melanoma, Maria Sklodowska-Curie National Research Institute of Oncology, 02-781 Warsaw, Poland; anna.janik@wum.edu.pl (A.M.J.); s080287@student.wum.edu.pl (A.T.); mateusz.spalek@pib-nio.pl (M.J.S.); bartlomiej.szostakowski@nio.gov.pl (B.S.); gp.chmiel@gmail.com (P.C.); klaudia.bobak@pib-nio.pl (K.B.); tomasz.switaj@nio.gov.pl (T.Ś.); piotr.rutkowski@nio.gov.pl (P.R.); 2 Faculty of Medicine, Medical University of Warsaw, 02-091 Warsaw, Poland; 3 Department of Radiotherapy I, Maria Sklodowska-Curie National Research Institute of Oncology, 02-718 Warsaw, Poland; 4 Department of Oncology, Oslo University Hospital, 0372 Oslo, Norway; kjetil.boye@rr-research.no; 5 Department of Pathology, Maria Sklodowska-Curie National Research Institute of Oncology, 02-781 Warsaw, Poland; anna.szumera-cieckiewicz@pib-nio.pl; 6 Diagnostic Hematology Department, Institute of Hematology and Transfusion Medicine, 02-776 Warsaw, Poland; 7 Department of Experimental Pharmacology, Mossakowski Medical Research Institute Polish Academy of Sciences, 02-106 Warsaw, Poland

**Keywords:** extrameningeal solitary fibrous tumor, EM-SFT, therapeutic strategies, soft tissue sarcoma, risk stratification, sarcoma

## Abstract

**Simple Summary:**

Extrameningeal solitary fibrous tumors (SFTs) are distinct mesenchymal neoplasms with the propensity for recurrence and the characteristic genetic marker of the *NAB2*-*STAT6* fusion gene. These tumors typically present as slow-growing masses, most commonly in the extremities or trunk. Different clinical behaviors ranging from low to high aggressiveness with the potential for metastasis can be observed. Diagnosis is based mainly on immunohistochemical staging for classic CD34 and STAT6 markers, although it can be perplexed by the similarity to other lesions. Surgical resection remains the primary treatment, and long-term follow-up is obligatory due to the unpredictable nature of SFTs. Further research is emerging to understand the biological behavior and optimal management with efficient treatment of SFTs.

**Abstract:**

Solitary fibrous tumors (SFT) are rare mesenchymal neoplasms that account for less than 2% of all soft tissue masses. In the latest WHO 2020 Classification of Soft Tissue Tumors, extrameningeal SFT was listed as intermediate (rarely metastasizing) or malignant neoplasms. Due to the lack of characteristic clinical features, their diagnosis and treatment remain challenging. The pathogenesis of SFT is often associated with the presence of fusions of the NAB2-STAT6 gene on the 12q13 chromosome. Cytoplasmic CD34 positive staining is considerably characteristic for most SFTs; less frequently, factor XII, vimentin, bcl-2, and CD99 are present. A key factor in the diagnosis is the prevalent nuclear location of STAT6 expression. Radical resection is the mainstay of localized SFTs. In the case of unresectable disease, only radiotherapy or radio-chemotherapy may significantly ensure long-term local control of primary and metastatic lesions. To date, no practical guidelines have been published for the treatment of advanced or metastatic disease. Classical anthracycline-based chemotherapy is applicable. The latest studies suggest that antiangiogenic therapies should be considered after first-line treatment. Other drugs, such as imatinib, figitumumab, axitinib, and eribulin, are also being tested. Definitive radiotherapy appears to be a promising therapeutic modality. Since standards for the treatment of advanced and metastatic diseases are not available, further investigation of novel agents is necessary.

## 1. Introduction

Solitary fibrous tumors (SFTs) are rare mesenchymal neoplasms that account for less than 2% of all soft tissue neoplasms [1]. Furthermore, they also have an age adjusted incidence rate of 0.061 per 100,000 people per year [2]. Most often manifesting in individuals during their fifth and seventh decades of life, they do not show a significant predilection for gender [3]. In 1931, Klemperer and Rabin described them in the pleura for the first time [4]. In 1942, Stout and Murray reported similar tumors and named them ‘localized mesothelioma of the pleura’ [5]. However, in 1951, Stout and Hamidi changed the name to ‘solitary fibrous tumors’ [6]. In 1942, Stout and Murray first described hemangiopericytoma (HPC) [6]. However, in 1991, Goodlad et al. published a large series of extrathoracic SFT [7]. 

For many years, HPC and SFTs represented two histological subtypes of a single neoplasm [8,9]. In the newest WHO 2020 Classification of Soft Tissue Tumors, extrameningeal SFTs have been separated [10,11] and are listed as intermediate (rarely metastasizing) or malignant neoplasms [11]. Approximately 15–20% of SFTs are malignant, and even benign SFTs have the potential for malignancy, which requires complete surgical resection as the preferred treatment option [12]. SFTs can sometimes be associated with symptoms such as hypoglycemia, osteoarthropathy, arthralgia, and clubbing [3]. 

Histologically, SFTs are characterized by spindle cells within a collagen stroma, often arranged in a whorled pattern. They are highly vascular and may undergo myxoid degeneration [3]. Identifying and treating SFTs can be challenging due to their similarity to other soft tissue tumors [13]. In terms of imaging features, SFTs are often incidentally discovered on radiography or CT scans, but their appearance on these modalities is nonspecific, requiring further investigation. They can exhibit variable density on CT scans, with hyperdense lesions having abundant collagen. The presence or absence of calcifications is not a distinguishing factor, and there is often overlap in the type of enhancement observed [3]. Benign SFTs have a local recurrence rate of 8%, while malignant ones can recur in two years in up to 63% of cases [3].

## 2. Epidemiology

The occurrence rate of SFTs is approximately 0.6 per million individuals per year for meningeal SFTs and approximately 0.4 per million for extrameningeal. SFTs are reported most frequently in adults with a wide age range (20–94) [14,15]. They are usually diagnosed in the fifth to seventh decade of life [16,17]. SFTs are not commonly found in pediatric patients, but cases in children [18,19,20,21,22,23,24] and infants [25,26] have also been described. Furthermore, tumors located in the pleura occur later in life, in contrast to other anatomical sites [27]. However, this could be influenced by a mix of delayed diagnosis and variations in published data [28]. Most reviews indicate that SFTs do not show any gender predilection [29,30,31]. However, tumors located in the larynx (ratio 6:1) [32] and the fat-forming variant (ratio 3:2) of SFTs [16,33] appear to occur in men more than in women,, while the locations of the liver [34], the oral cavity [15], and the superficial localizations [35] show a slight female predominance. Interestingly, no association with asbestos exposure or smoking has been demonstrated [16]. Thus, the exact etiology remains unknown. Tumor size at presentation is very variable and depends on location. It ranges from 0.7–42.5 [36], and the median size is observed between 5–10 cm [27,34].

## 3. Anatomical Location

SFTs occur at almost every anatomical site and organ [37]. One of the largest analyses of 219 patients showed that the most common site for SFT development was the thoracic cavity, followed by the abdominal cavity [16,36]. Furthermore, most intrathoracic tumors originate in the pleura [17,38]. Approximately 5–27% of SFTs occur in the head and neck region [39] with the oral cavity [40] and the orbit [39] being the most commonly affected sites. A few studies showed that pelvic [27,30,41], abdominal [27,42], and pleural [27,40,43] SFTs tend to be larger than tumors appearing in other locations, and extrapleural SFTs may show potentially malignant behavior [44], especially those with limb localization [45]. Generally, over the years, SFTs have been described in various localizations, including the liver [46,47], kidney [48,49,50], pancreas [23,51,52], sigmoid colon [31], mesocolon [14], omentum [53,54], urinary bladder [55], caecum wall [56], mesorectum [41], prostate [57], spermatic cord [58], testis [59], scrotum [60], female gynecological tract, especially the vulva [61,62], vagina [63], breast [64,65,66], heart and pericardium [33,67,68], epicardium [69], pulmonary artery [70], trachea [71], larynx [32,72], tongue [15], salivary glands [73,74,75,76], sinonasal and rhinopharyngeal tracts [77], external auditory canal [78], orbit [79,80], adrenal glands [24], thyroid glands [81,82,83], extremities [27,84,85,86,87], chest [88], subcutaneous regions [89], and superficial area [35,90]. 

## 4. Diagnosis

SFTs remain a diagnostic challenge due to the lack of characteristic clinical features [91]. They mostly occur in the form of benign [31], slow-growing [14], asymptomatic tumors [14,52] incidentally diagnosed during routine health screening [54,92] or imaging while investigating other pathologies [41,46,93,94]. If symptoms are present at admission, they tend to be local and unspecific, and reflect mass-related compression of adjacent structures in about 90% of reported cases [17,80,86,95,96,97]. Moreover, most symptomatic cases present with pain [31,98,99,100], and these tumors tend to be larger than asymptomatic ones [82]. Depending on the location, other manifestations include obstructive jaundice [52], a burning sensation during urination [14], urinary retention [57], abdominal discomfort [30], nausea, vomiting [48], hematochezia [31], hematuria [91], swelling [18], proptosis, blepharoptosis [18], dyspnea [33,69,98], sexual disorders, difficulty with defecation [91], coughing [98], and chronic bronchitis [33]. Furthermore, approximately 63% of SFTs located in the sinonasal tract present with nasal obstruction, and 87% of laryngeal tumors cause dysphonia [101]. In some cases, systemic symptoms such as arthralgia, hypertrophic osteoarthropathy, or clubbing occur [102,103]. Moreover, a small percentage of patients present with the paraneoplastic syndrome of hypoglycemia (<5% patients) [102,103] known as “Doege-Potter syndrome” [37] or acromegaly changes [104]. This is due to the tumor’s excessive secretion of insulin-like growth factor [105,106]. This is more common in tumors of the retroperitoneum and liver [107,108] and has not been reported among patients with SFTs localized in the head and neck region [109]. Hypoglycemia is associated with weight loss, fatigue, night sweats, or unsteady gait [98]. Furthermore, it is considered a poor prognostic indicator because, in approximately 70% of cases, tumors present malignant behavior [38,110]. However, all systemic symptoms could resolve after resection of the mass [46,111,112,113,114,115].

**Figure 1:**
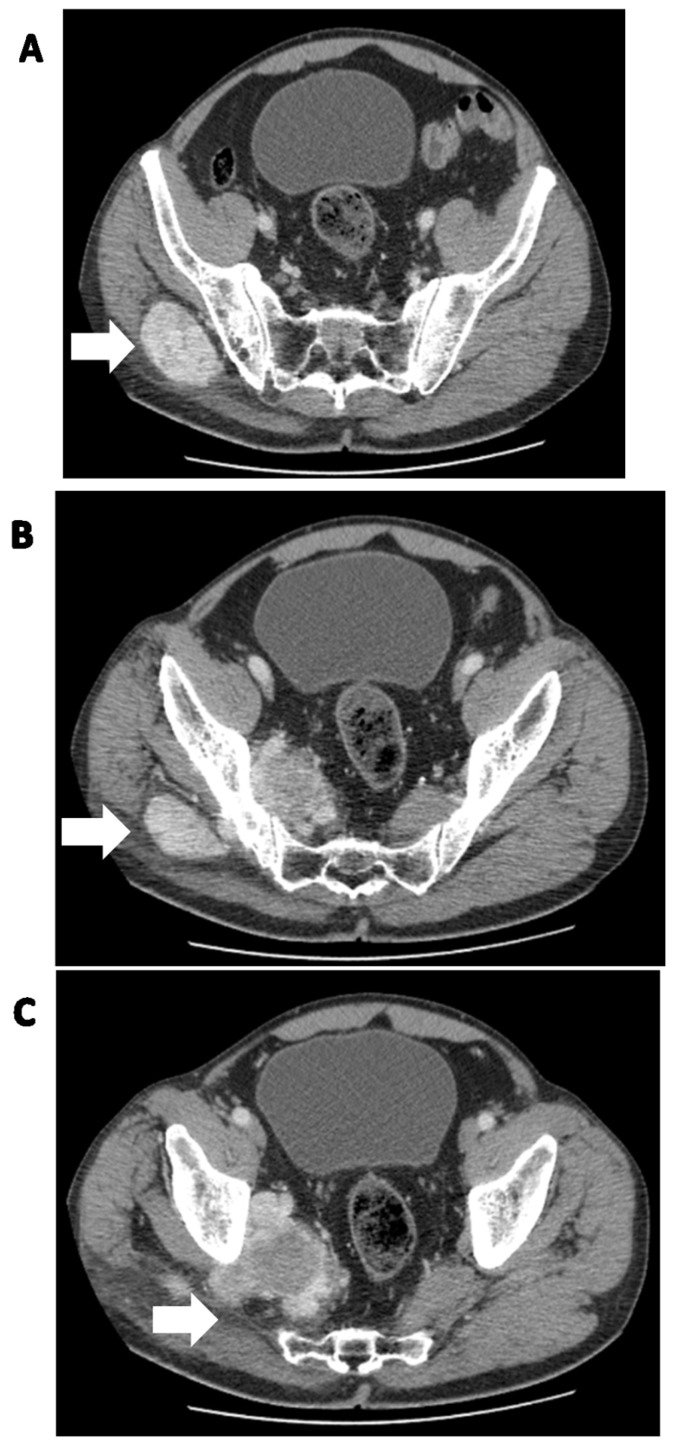
Solitary fibrous tumor of the pelvic region in a 62-year-old man. (**A**) Soft tissue mass in the right iliac region (white arrow), (**B**,**C**) passing through the pelvic obturator opening into the small pelvis (white arrows).

**Figure 2:**
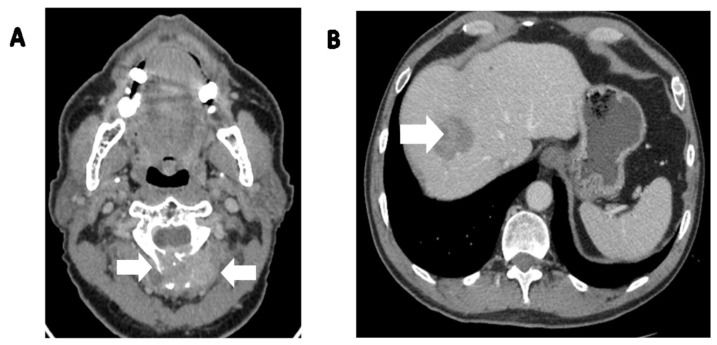
Synchronous metastatic lesions in the same patient as in Figure 1. (**A**) Metastatic lesion (white arrows) with destruction of the C3 vertebra and soft tissue involvement; (**B**) metastatic liver lesion (white arrow).

Preoperative diagnosis by imaging of both pleural and extrapleural SFTs is challenging because findings are unspecific [30,47]. Although it is impossible to distinguish benignly behaved tumors from malignant ones [16,47] or to differentiate SFTs from other mesenchymal tumors [53] or spindle cell tumors [116], imaging findings can provide important information, especially before resection. The diagnostic modalities of choice are computed tomography (CT) scanning and magnetic resonance imaging (MRI) (Figure 1 and Figure 2) [53]. CT provides basic information about tumor size, morphology, and location, and determines its relationship to surrounding organs [33]. In less than 5% of patients [17], MRI shows the presence of hemorrhage, necrosis, and degeneration [33] which occurs in larger and malignant tumors [17]. Additionally, MRI provides information about vascularity [33] and helps to identify fibrous content [16]. It also excludes local invasion of close structures [98]. On CT, SFTs appear as solitary, well-defined, ovoid, rounded, or lobulated solid masses [54], sometimes with regions of punctuated calcification [17]. The effect of enhancement on CT or MRI is variable [17,86] and depends on the tumors’ vascularization [98]. MRI shows SFTs as isointense on T1-weighted images and variable on T2-weighted images, often described as a black-and-white-mixed pattern [3]. Ultrasound typically depicts SFTs as hypoechoic or heterogeneous lesions, with the latter corresponding to areas of myxoid degeneration. In PET-CT scans, benign SFTs exhibit low-grade activity, while malignant SFTs tend to be strongly hypermetabolic and homogeneous. Lesion multiplicity and adjacent rib destruction can be indicators of malignancy, even though benign SFTs can also cause rib destruction [3]. SFTs in different locations within the body may have distinct imaging characteristics. Imaging modalities such as CT, MRI, ultrasound, and PET scans aid in diagnosing and characterizing these tumors, but the definitive diagnosis relies on histological examination [3].

Interestingly, FNA (fine needle aspiration), together with nuclear STAT6 immunoreactivity, has been reported to be an additional tool in the diagnosis of SFTs [117]. However, a core needle biopsy or an open incisional biopsy [118] and an immunohistochemical analysis are needed to confirm the diagnosis [55]. In STS, an open incisional biopsy may comprise treatment due to tumor dissemination, and as such could only be indicated in singular cases, e.g., in unfeasibility of a core needle biopsy [119]. However, indications for open biopsy should be determined in a reference centre [120]. The metastatic rate of SFTs is up to 35–45% [121]. Furthermore, tumors classified primarily as benign may show malignant behavior many years after initial diagnosis [122]. For this reason, patients require long-term observation [29,44,100,123], especially those with tumors greater than 10 cm [16,124]. Metastases are most commonly described in the lungs [27,124], liver, and bones [27,107,124]. Furthermore, metastases have also been reported in subcutaneous tissue [44], adrenal glands, brain, muscles [17], and kidneys [27]. Interestingly, a case of pulmonary carcinoid metastasized to an intraparenchymal SFT in the same lung lobe has been described [125]. Furthermore, abdominal SFTs have a higher rate of metastasis to multiple organs outside the peritoneum, while pleural tumors metastasize more frequently locally to the hemithorax [27].

## 5. Clinical and Radiological Differential Diagnosis

**Figure 3:**
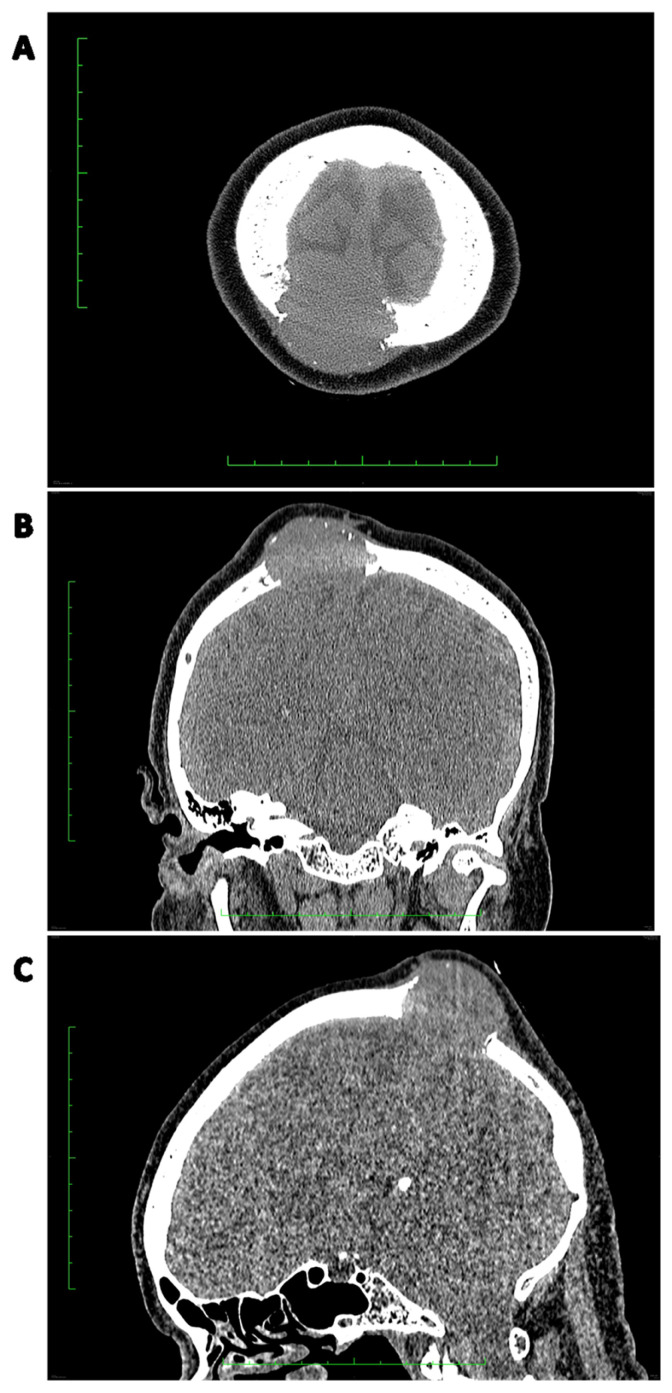
A 65-year-old patient with locally advanced SFT in parietal bone with infiltration of the superior sagittal sinus with adhesion to the dura mater was admitted in 2022 to begin systemic treatment. Symptoms had begun in March 2019, with a growing subcutaneous tumor in the parietal area. It was initially excised by a dermatologist and diagnosed as lipoma. In February 2022, due to tumor recurrence, the patient underwent a biopsy which was diagnosed as a malignant solitary fibrous bone (SFTB). (**A**) CT scan, axial view showing the destruction of the parietal bone. (**B**) CT scan, coronal view showing bone destruction with a soft tissue mass adhering to the skin. (**C**) CT scan, sagittal view clearly depicting the size of the tumor in relation to the parietal bone and skin.

Clinical and radiological differential diagnosis is challenging, and depends on the location of the tumors (Figure 3). An accurate diagnosis is essential because some cancers can be treated with chemotherapy [41]. The following are taken into account: neuroendocrine tumors, solid pseudopapillary cancers, hemangiomas [52], haemangiopericytoma, schwannoma [126] leiomyosarcoma, lymphoma, histiocytoma [91], cholangiocarcinoma, synovial sarcoma, dermatofibrosarcoma protuberans, oncocytoma [127], diffuse malignant mesothelioma [128], juvenile paraganglioma, angiofibroma, smooth muscle neoplasms [129], other spindle cell tumors [130], fibrosing hamartoma, fibromatosis [7], intestinal invagination, stromal tumor [56], nerve-sheath tumors [89], and anaplastic and papillary carcinoma [81]. Generally, all the neoplasms have a dominant fibrous part or high vascularization [91]. A rare occurrence in the thyroid made it perplexing to distinguish SFT from thyroid spindle cell lesions, mainly due to the application of FNA in diagnosing these organ neoplasms. SFT does not harbor specific cytological features, thus deep specimen evaluation and immunohistochemistry are recommended [131].

## 6. Pathology

### 6.1. Histology

**Figure 4:**
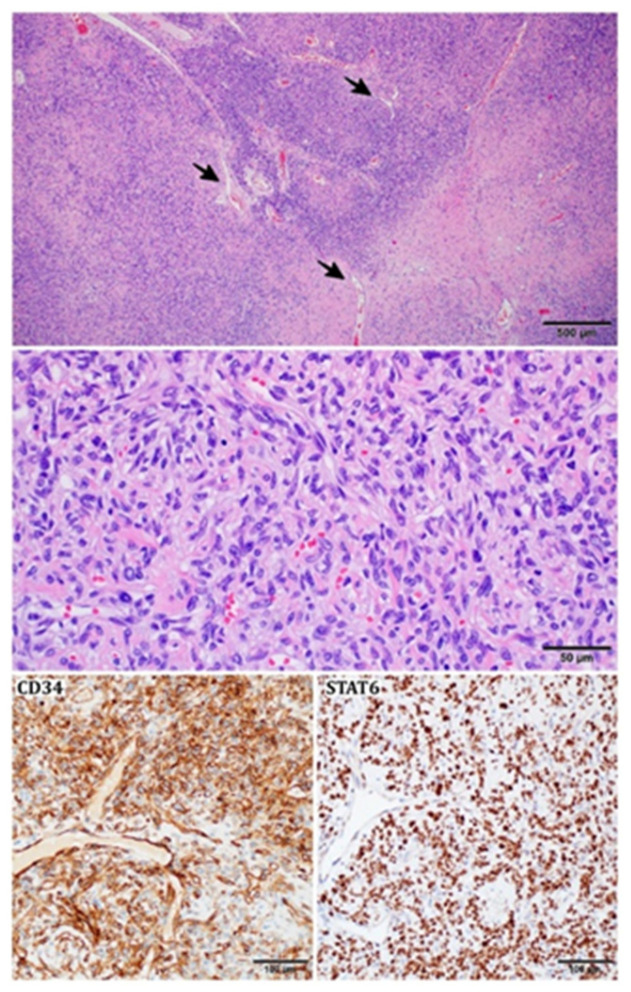
Histopathological image of an SFT: variable cellularity with prominent “staghorn-shaped”, branching vessels (black arrows); tumor cells are spindled or ovoid; stroma is focally collagenous; immunohistochemically SFT shows strong expression of CD34 and nuclear STAT6.

Microscopically, in SFTs, ovoid and fibroblastic spindled cells are randomly distributed, interlacing numerous dilated pericytomatous vessels. Hyalinization fields and collagen fibers are present, with the latter defining the varying cellularity of specific areas [16,73,132,133,134]. Cells present with scant, light cytoplasm (Figure 4) [135]. Myxoid changes are typical for many SFTs; however, their dominance is rarely described; when they occur, individual cell agglomerates may be visible, and the SFTs are susceptible to false identification [136]. Defining the mitotic number may often pose a challenge [16]. The rare appearance of necrosis, cell pleomorphism, hypercellularity, infiltration in the periphery, and a mitotic count of at least 4/10 HPF define the malignancy of SFTs and the probability of metastases [27,31]. The Ki-67 labeling index has also been deemed adequate as a prognostic factor in risk models [137]. 

### 6.2. Immunohistochemistry 

Cytoplasmic CD34 positive staining is considered characteristic for most SFTs [138,139], as well as factor XII, vimentin, bcl-2, and CD99, with less regular presence of the latter [73]. Positive staining for NF68 and neuron-specific enolase has been described in some SFTs [138]. Most SFTs are negative for CD31 [138], CD68 [73], CD117, inhibin, smooth muscle actin (SMA), desmin [31], cytokeratin, S-100 protein [140], chromogranin, and synaptophysin [138]. SMA-positive staining has been reported to be present in two cases of oral soft tissue SFT [73]. Furthermore, in some cases of actin positivity in SFTs, it has been attributed to the differentiation of neoplastic cells into myofibroblasts [135]. Singular cases of desmin-positive and cytokeratin-positive staining in SFT cells have been described [138]. A recent study suggested the presence of neuroendocrine markers in SFT, such as INSM1 (positive in 35.7%), synaptophysin, CD56, and CD57. However, no expression of chromogranin was observed [141]. The specificity of NAB2-STAT6 fusion allows the usage of STAT6 immunochemistry (IHC) in the identification of SFTs (e.g., fat-forming), which may otherwise prove problematic in differentiation [142]. A key factor in diagnosis is the prevalent nuclear location (rather than cytoplasmic) of STAT6 expression [16]. The stains mentioned above (CD99, CD34, bcl-2) were described to be less distinct for SFTs than STAT6 [134]. GRIA-2 immunochemistry is equivalently useful in distinguishing SFTs; however, due to the variety in STAT-6 and GRIA-2 expression patterns in SFT mimics, both markers should be considered, as their complementarity is utilized [143].

### 6.3. Lymphocytic Infiltration 

Lymphocytic infiltration is a feature found in some SFTs, e.g., extrathoracic ones [108]. Cases of head and neck tumors in which these infiltrations produced nodules were also reported [129]. In both classical and highly malignant SFTs, T cells are relatively infrequent. IHC, in general, suggests some suppression of immunological components. Antiangiogenic agents can intensify lymphocytic infiltration, suggesting the importance of immunological constitution in such treatments [144]. 

### 6.4. Differential Diagnosis 

CD34 staining allows the differentiation of SFT from desmoplastic mesotheliomas [145]. In the differential diagnosis of tumors in the upper extremities, the presence of cells positive for desmin distinguishes tumors from desmoid. At the same time, actin-positive staining and myxoid parts represent nodular fasciitis [140]. When SFTs differentiate from immunohistochemically similar tumors, STAT6 positive staining and the specific pattern of the vessels are crucial [146].

### 6.5. Genetics 

The cornerstone of SFT pathogenesis is linked to the presence of NAB2-STAT6 gene fusion from the 12q13 chromosome [16]. Gene fusions with STAT6 modify the function of the transcriptional repressor NAB2, leading to the activation of EGR1-oncogenic gene targets and the initiation of sarcomagenesis [147]. Although NAB2-STAT6 is pathognomonic for SFT, the exact percentage of tumors with confirmed fusion differs among researchers, with some pointing to 100% of examined tumors [148], others to 91% [149], and some even to just above half of the cases [150], leaving room for further comparison of genetic diagnostic techniques. This genetic trade has been implicated as the central factor in SFT formation, making the inhibition of EGR targets worth exploring as a treatment option [148,150]. Forty or more breakpoint variants involving different exons of the fusion partners have been described. The most prevalent fusion variant is NAB2ex4-STAT6ex2, followed by NAB2ex6-STAT6ex16 and NAB2ex6-STAT6ex17 [151,152]. The different NAB2-STAT6 fusion variants are associated with distinct clinicopathological characteristics and transcriptional signatures, and have prognostic significance [153,154]. NAB2-STAT6 fusion variants exhibit an age-dependent pattern, as well as a correlation with specific tumor locations and mitotic rates. NAB2ex4-STAT6ex2/4 is, for example, present in SFTs with low mitotic rates, in older patients, and most commonly in tumors of the intrathoracic region [152]. In dedifferentiated SFT cases, STAT6 expression may diminish, but the fusion can still be identified through PCR testing. While the specific fusion transcript’s impact on prognosis remains unclear, other molecular factors like TP53 or TERT (telomerase reverse transcriptase) mutations have been linked to a poorer prognosis [155]. Notably, the length of the STAT6 gene significantly influences clinical outcomes. Patients with STAT6-TAD (fusion with the transactivation domain of STAT only) have tumors with significantly increased mitotic count and high recurrence risk, contrary to patients with STAT-Full (fusion with most domains of STAT). Ten-year estimated recurrence-free survival in the STAT-Full cohort was 78%, and in the STAT-TAD group only 25% [153]. Research indicates that further examination of particular NAB2-STAT6 fusion variants could significantly improve the stratification of patients, especially in the intermediate-risk group [123,156]. Next-generation sequencing subtype, targeted RNA-fusion sequencing (e.g., the FusionPlex Sarcoma Panel—Archer Dx), may be a promising option for detecting fusion variants in diagnostically demanding patients [149]. 

Furthermore, all SFTs have an overexpression of at least one kinase, including EGFR, ERBB2, FGFR1, JAK2, or DDR1, as well as deregulation of retinoic acid receptors and histone deacetylases [157,158]. SFTs are also characterized by a significantly upregulated stem cell marker (ALDH1), which could become a new diagnostic marker [157]. The AURKA gene has been mentioned as a potential prognostic factor [157]. Overexpression of IGF2, associated with loss of imprinting, was consistently present in all SFTs [158]. Interestingly, upregulation of the kinases mentioned above, the receptors, deacetylases, and IGF2 is independent of the anatomical location of the tumor, in contrast to IGF1 and JUN which are found only in pleural tumors [157,158]. The GRIA2 gene is also significantly overexpressed in most SFTs. Therefore, it is used as a marker to differentiate these tumors from similar soft tissue neoplasms [143]. However, not only were single gene mutations or expression dysregulation reported, but also loss of 13q and 14q chromosomes [147]. Evaluation of copy number variations (CNV), including gain of the eighth chromosome, may be of use in evaluating the malignant potential of SFTs, as its presence has been shown mainly in larger tumors (>10 cm) with high mitotic rates [159]. Reactivation of TERT due to its promoter mutations plays a significant role in the pathology of SFTs, and is considered a prognostic biomarker [160]. These genetic variations have been associated with shorter time to first metastasis, larger tumor size, worse event-free survival, older age, and higher-risk classifications; however, their predictive value in clinical outcomes and overall survival is still being discussed [160,161]. The TERT promoter mutations are important for risk assessment in patients with intermediate-risk tumors [161]. Furthermore, in the case of pleural SFTs with paraneoplastic syndromes, BRCA1 mutations were implicated in the pathogenesis [162]. Furthermore, novel studies indicated the role of epigenetic changes in SFT. The methylation profile is highlighted in current algorithms for SFT classification. However, the latest preliminary data indicate that it may be inaccurate. For example, high precision in classification based on methylation profile was shown in intracranial tumors, whereas in soft tissue or bone SFT it utterly failed. Therefore, SFT classification schemes using gene methylation profiles could be deficient and require further analysis, taking into account the heterogeneity of SFTs [157,163].

## 7. Treatment of Localized Disease

### 7.1. Surgical Resection 

**Figure 5:**
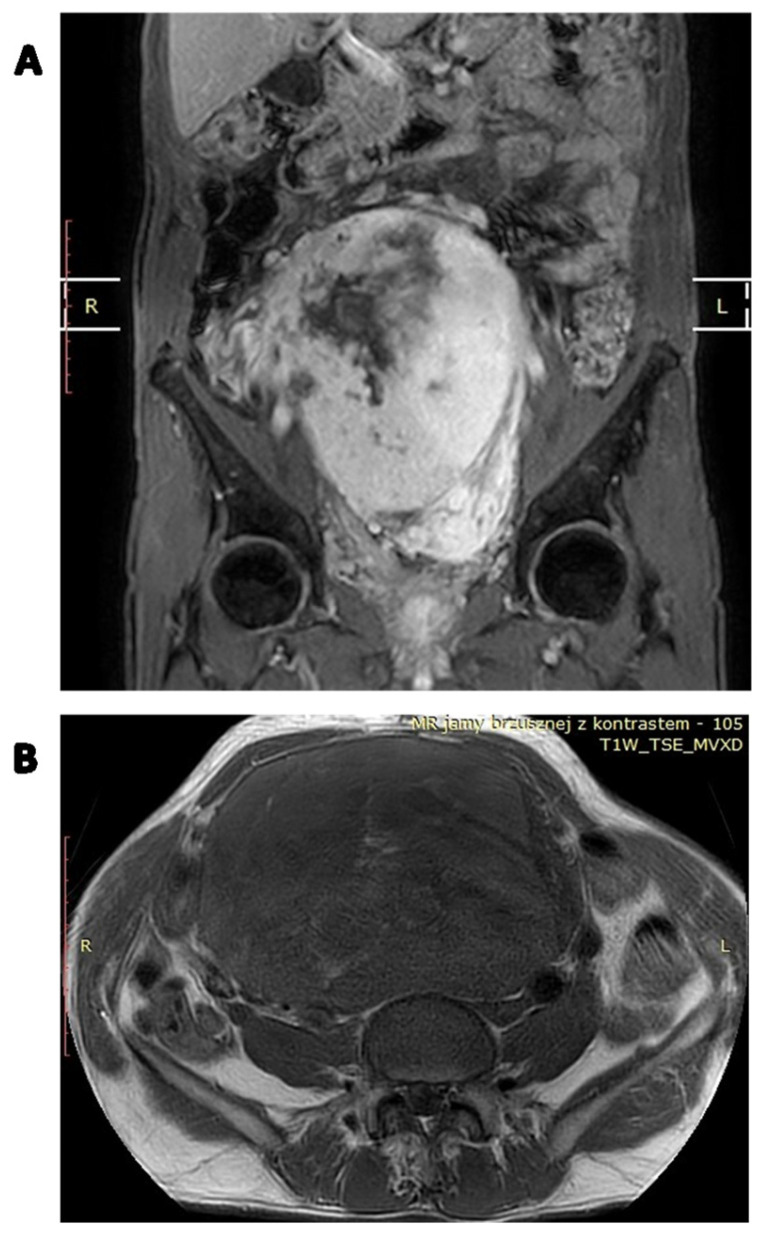
Contrast-enhanced MRI image of a locally advanced abdominal SFT: (**A**) coronal image; (**B**) axial image.

**Figure 6:**
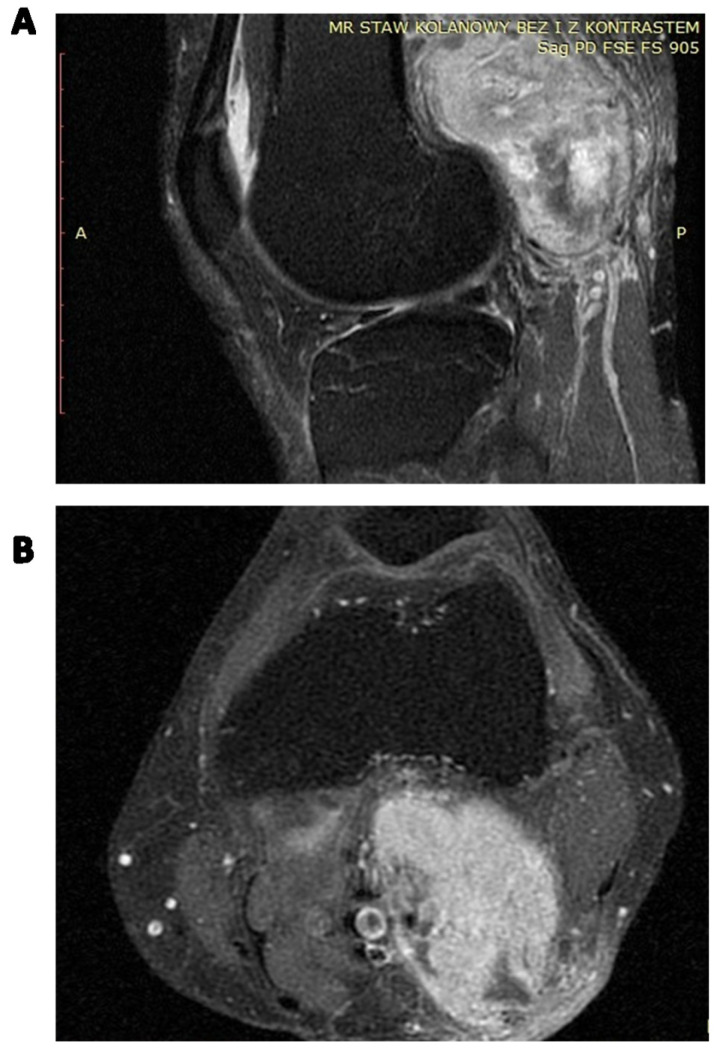
Contrast-enhanced MRI of the locally advanced left popliteal fossa SFT abutting popliteal vessels: (**A**) coronal image; (**B**) axial image.

Localized SFTs account for more than 90% of all SFT cases [164], therefore radical resection is the mainstay of treatment (Figure 5 and Figure 6) [128,165,166,167]. In a retrospective study of 549 patients, 428 (78%) underwent surgery alone and 121 (22%) underwent surgery together with postoperative RT. Surgical margins: R0 and R1 were achieved in 73.6% and 22.6% of the patients, respectively [166]. In another study, negative margins were achieved in 94.44% of patients who underwent surgical resection [13]. In addition, video-assisted thoracic surgery can be performed in the case of thoracic tumors less than 10 cm in diameter [168]. Furthermore, embolization with prior angiography is recommended in tumors attached to the visceral pleura. It is the most efficient procedure that reduces bleeding during the operation [169]. Resection of pelvic SFTs can be associated with a high risk of bleeding; therefore, balloon blockage of the aorta is a good prevention option [170]. In addition, successful laparoscopic excisions have been performed on benign tumors located in the abdominal cavity or pancreatic head. In both cases, no relapse was found during the 20- and 6-month follow-up, respectively [171,172]. Additionally, robotic operations have been implemented in SFTs with efficacy [173]. There are no exact data on the size of resectable tumors. Therefore, preoperative tumor evaluation is essential to select the best surgical access [174]. Pulmonary wedge excision, lobectomy, or segmentectomy can be performed for malignant tumors or local relapse [128]. In addition, where local relapse has occurred, multiple reoperations may be considered [128,174]. 

### 7.2. Perioperative Radiotherapy 

**Figure 7:**
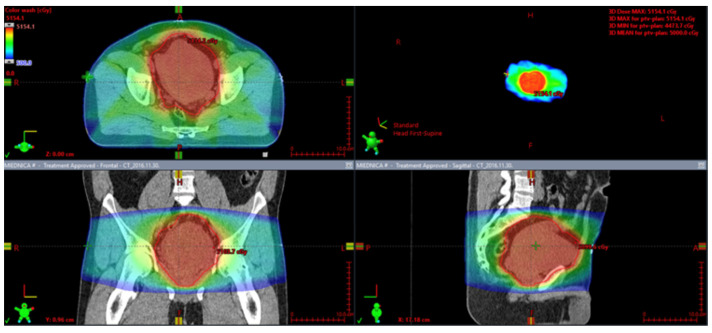
Preoperative radiotherapy for a marginally resectable pelvic solitary fibrous tumor.

**Figure 8:**
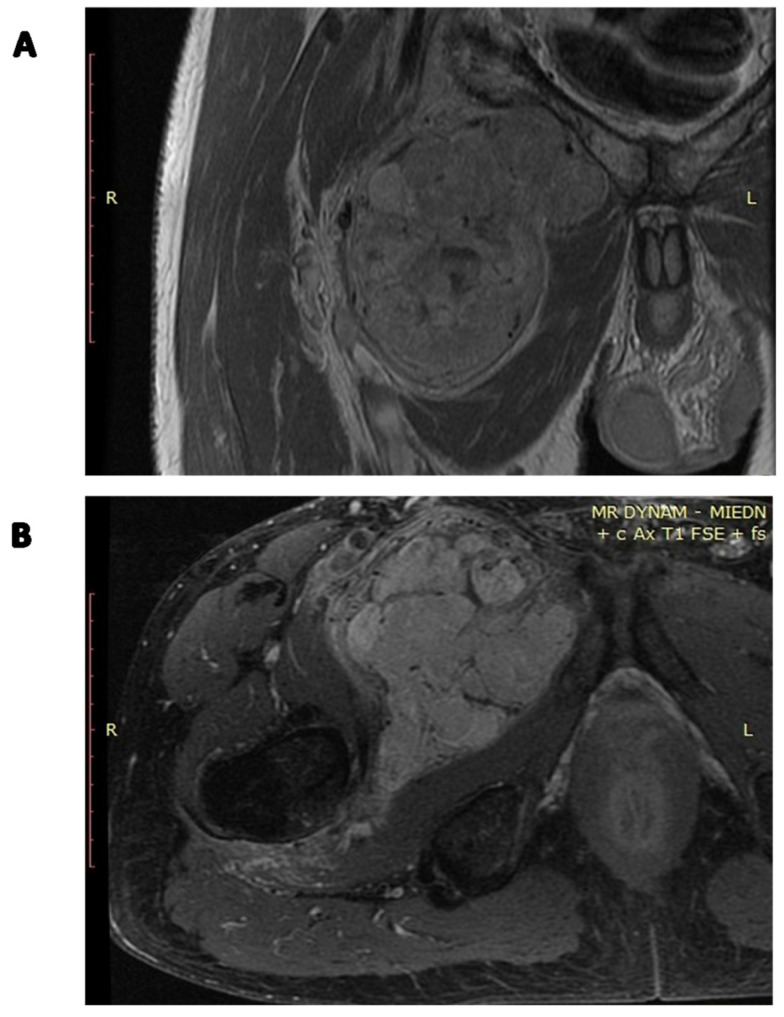
Preoperative radiotherapy contrast-enhanced MRI image of the SFT of the right groin: (**A**) coronal image; (**B**) axial image.

The role of perioperative radiotherapy in localized SFTs was the subject of some retrospective studies. A large retrospective study of 549 patients with localized and resectable SFTs showed that surgery in combination with preoperative or postoperative radiotherapy in a total dose of approximately 50 Gy reduced the risk of local relapse (*p* = 0.12), especially in tumors with high mitotic count and unclear surgical margins. However, irradiation did not influence overall survival [166]. This analysis suggests that perioperative radiotherapy should be considered in a selected group of patients, namely those with intermediate or high-risk tumors based on the Demicco risk stratification scoring system [166]. Krengli et al. also showed that additional adjuvant radiotherapy, compared to surgery alone, improves the local control rates and disease-free survival, especially if tumors are located in the extremities or superficially in the trunk (LC = 91.6%, *p* < 0.0001 and DFS = 83.1%, *p* = 0.008) [175]. (Figure 7 and Figure 8).

Moreover, in the case of mediastinal SFTs with external invasion, postoperative radiotherapy may provide a substantial benefit in local control [174]. Some studies report a regain of resectability after preoperative radiotherapy [176,177]. Therefore, SFTs may not be as radioresistant as other soft tissue sarcomas. An example of preoperative radiotherapy in a marginally resectable pelvic SFT is presented in Figure 4. The patient received 50 Gy in 25 fractions.

### 7.3. Perioperative Chemotherapy 

The efficacy of chemotherapy on SFTs is limited [47]. Neoadjuvant chemotherapy is generally not recommended due to poor effectiveness and the uncertainty of impact on overall survival (OS) [178]. However, there are examples of successful preoperative systemic treatment. On the contrary, neoadjuvant doxorubicin chemotherapy remained ineffective [44]. Interestingly, in a case of SFT localized in the pulmonary artery, adjuvant 21-day chemotherapy was prescribed [70]. The patient received 2.0 g of ifosfamide, i.v. day 1–3, and 100 mg epirubicin civ (continuous intravenous) 96 h. Stable disease (SD) was achieved. In the next phase, a 250 mg apatinib per day was added. Two years later, the patient remained alive [70]. In 2021, Zhi-Ke Li et al. published a case report of a patient with a locally advanced, malignant, unresectable solitary fibrous tumor that was primarily misdiagnosed and treated as Ewing Sarcoma [179]. The patient received eight cycles of chemotherapy: 2 mg vincristine (day 1), 120 mg doxorubicin (day 1) plus 2 g cyclophosphamide (day 1)/3 g ifosfamide (day 1–5) plus 150 mg etoposide (day 1–5), every three weeks. A partial response was achieved, and the tumor reduced in size, so surgical resection was performed. In addition, he received adjuvant radiotherapy (56 Gy in 28 fractions of 2 Gy each). In one year of follow-up, neither relapse nor dissemination was observed [179]. Although treatment was effective in these patients, the treatment strategy of chemotherapy followed by surgery and radiotherapy needs further investigation. Interestingly, preoperative apatinib treatment was performed on a tumor involving the pelvic ring and sacrum, and partial response (PR) was achieved [170]. The latest trial suggests that pazopanib treatment has a potentially beneficial effect on unresectable malignant SFTs [164], but these studies require further validation.

### 7.4. Definitive Radiotherapy

In cases of unresectable disease, only radiotherapy or radio-chemotherapy may significantly ensure long-term local control of primary and metastatic lesions. A large multicenter study of patients with localized, unresectable, or locally recurrent tumors showed that radiotherapy alone in a total dose of 60 Gy provided acceptable long-term local control. Complete response (CR), PR, or SD was achieved in a total of 93% of the cases with 87.5% five-year overall survival. Furthermore, tumor size did not have any effect on treatment outcomes [180]. Vanfleteren et al. published a case report of an unresectable SFT localized in the mediastinum treated with chemo-radiotherapy [181]. The patient received three cycles of chemotherapy with cisplatin (AUC 5 mg/mL/min), later replaced with carboplatin due to low creatinine clearance, and etoposide (100 mg/m^2^) together with 42 Gy radiotherapy. All symptoms of the disease resolved and the tumor shrank, but it was still unresectable. At eight months of follow-up, the patients were able to lead an active life [181]. The report shows that the systemic treatment strategy may be taken into account in the case of symptomatic unresectable tumors to alleviate symptoms. Another report presented a case of a patient with unresectable recurrent malignant intrathoracic SFT [182]. The patient received 50 Gy in 25 fractions followed by a boost of 10 Gy in five fractions for a 22 cm tumor in the left thorax. Treatment was well tolerated. The authors reported a significant PR and long-term local control. Heavy-ion therapy was effective in treating recurrent malignant spinal SFT [183]. Particle therapy should be considered in tumors that arise from challenging localizations. However, more research is needed.

## 8. Treatment of an Advanced and Metastatic Disease 

**Figure 9:**
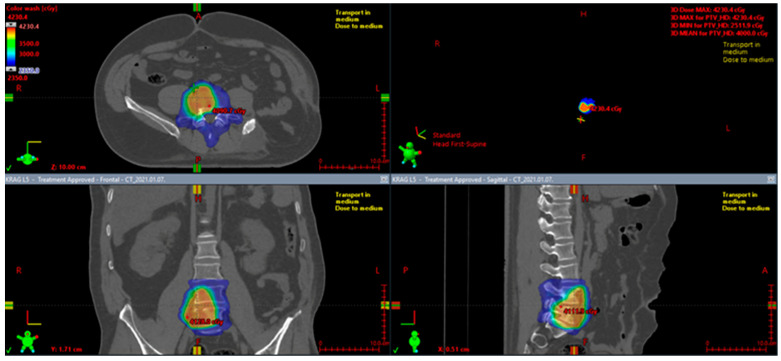
Stereotactic radiotherapy for a metastatic solitary fibrous tumor.

Treatment of metastatic disease is very challenging. To date, no clear guidelines have been published [167], while in our clinic all patients are subject to a multidisciplinary team decision according to national treatment guidelines [184]. Most commonly, anthracyclines are used in the first line of treatment, while trabectedin, dacarbazine, and ifosfamide are used in subsequent lines [167]. Doxorubicin monotherapy is the preferred first-line treatment [167]. In selected cases in the first line of treatment, the combination of doxorubicin and dacarbazine is preferred [185]. Furthermore, an objective response has been reported in patients with recurrence and lung metastasis treated with doxorubicin and gemcitabine in combination. In this case, radiological follow-up showed PR [48]. In one of the analyses of advanced unresectable metastatic tumors or potentially resectable cases, patients received chemotherapy regimens based on doxorubicin or gemcitabine [186], but an objective response was not achieved. However, in 89% of the participants, SD was reported [186]. In another study of locally advanced or metastatic cases, patients received anthracycline monotherapy or in combination with ifosfamide [187]. In 20% and 27% of the cases, PR/SD were observed, respectively. However, the rest of the tumors progressed [187]. Comparable results were obtained in other analyses, with doxorubicin, ifosfamide, palifosfamide, brostallicin, vinorelbine, and paclitaxel alone or in combination with carboplatine. Median progression-free survival was 5.2 months [188]. Stacchiotti et al. analyzed 12 patients with advanced (malignant or dedifferentiated) SFTs who received combined treatment based on doxorubicin (75 mg/mq, iv bolus) and dacarbazine (800 mg/mq, intravenously over 60 min, two days), every three weeks. The median OS and PFS were 19 months (range 9–44+) and six months (range 2–32), respectively. Furthermore, the median PFS for malignant tumors was six months, while for dedifferentiated SFT it was four months longer [189]. On the contrary, De Vito et al. showed that patients with primary metastatic disease have worse OS and conventional chemotherapy is not associated with long-term positive effects [190]. Furthermore, surgical cytoreduction in combination with hyperthermic intraperitoneal chemotherapy has been tested in patients with recurrent SFTs with liver dissemination. Subsequently, the patient developed lung metastases and was treated with palliative chemotherapy to achieve SD [191]. At the same time, a valuable method for the management of metastatic SFTs could be high-dose ablative radiotherapy or metastasectomy, as shown by multiple sarcoma centers. Two recent studies analyzed large cohorts of patients with soft tissue and bone sarcomas who were treated with stereotactic radiotherapy (Figure 9). These groups included 10 patients with SFT [184,192]. Both studies confirmed the high local efficacy of stereotactic radiotherapy. An example of stereotactic radiotherapy administered for a metastatic SFT is presented in Figure 5. The patient received 40 Gy in 8 Gy fractions for the gross tumor volume and 25 Gy in 5 Gy fractions for the elective volume of the affected vertebrae. In cases progressing with metastases after excision of the primary tumor, metastasectomy may be performed [168,193]. However, this carries a risk of death within months, essentially if no subsequent adjuvant systemic therapy is used [168]. A case of a patient with a primary SFT located in the abdominal cavity and multiple liver metastases treated with surgery and radioembolization has been reported [194]. Palliative radiotherapy for metastatic disease with a total dose of 39 Grays was shown to be beneficial. Haas et al. showed that it positively affects both the five-year local control and OS at 62.5% and 54.2%, respectively [180]. 

## 9. Antiangiogenic Treatment 

The latest studies suggest that antiangiogenic therapies should be considered after first-line treatment, with the exclusion of dedifferentiated SFTs [121]. Pazopanib and sunitinib are effective against SFT [188,195]. In a recent study of typical advanced SFTs, pazopanib has been labeled the best first-line treatment for this type of tumor, with partial responses according to Choi criteria in 58% of patients and stable disease in 39% (RECIST: 94% of patients with stable disease and partial response in 6%) [164]. Other studies suggested that bevacizumab and temozolomide are efficient in SFT treatment (PFS of 9.7 months, the highest of all investigated antiangiogenics) [196]. However, in these studies, a patient selection bias has occurred [121]. Some authors indicated that this combination is equally as active as temozolomide alone [197]. Sunitinib therapy has been described to provide PFS of six months; however, in some patients, long-term answers have been achieved [198]. Sunitinib therapy results most often in SD by RECIST [197] and decreases tumor density [198]. In another study, dacarbazine and temozolomide were shown to be more active against SFT than pazopanib, bevacizumab, and sunitinib. Sorafenib is also likely an active agent in SFT treatment with OS similar to other angiogenic agents, but more research is needed [199]. Recently, a phase II trial with regorafenib was conducted in adult patients with advanced and progressive SFTs (EudraCT number: 2015-002629-21). In the group of 16 enrolled patients, the results incorporated one PR, 12 SD, and three PD; however, in the dedifferentiated subtype, no response was achieved. Furthermore, multiple-dose reductions were observed [200]. 

## 10. Other Treatment Possibilities

According to Yamada et al., between 50 and 80% of SFTs are positive for mTOR, p-Akt, and S6RP; therefore, activation of the Akt-mTOR pathway correlates with tumor malignancy. At the same time, receptor tyrosine kinases (RTKs), such as IGF1R and PDGFR-B, exhibit high levels of phosphorylation in SFTs, and are possibly linked to activation of the Akt-mTOR, MAPK, and JAK/STAT pathways [201]. The case study by Prunotto et al. showed that in malignant SFTs imatinib could be considered as a possible targeted therapy, as platelet-derived growth factor receptor beta (PDGFR-B) is overexpressed and phosphorylated in SFT cells, while imatinib inhibits its phosphorylation, and simultaneously decreases the expression of alpha SMA, resulting in a reduction in cell proliferation and differentiation [202]. Figitumumab and IGF1R inhibitors (insulin-like growth factor 1 receptor), in general, seem potentially successful in the treatment of SFTs, with limited side effects, in patients with a specific signaling profile, and it is an area that needs to be further explored in the future [203]. A study in 2019 shows that for progressive advanced SFT, axitinib is active with Choi-ORR of 54% (considering only malignant SFTs), and a median Choi-PFS of 5.1 months (14.8 months in responsive patients only). Promisingly, this treatment is effective in patients with resistance to other antiangiogenic drugs [204]. Eribulin activity in advanced SFTs is now being investigated in an ongoing phase II trial NCT03840772 (https://clinicaltrials.gov/ct2/show/NCT03840772, accessed on 15 September 2023).

## 11. Risk Stratification Models of Poor Outcomes 

### 11.1. Factors Predicting Local Recurrence

The overall recurrence rate differs from 10–40% [26,27,45,167,205,206]. The incidence of local relapse increases significantly after 20 years from diagnosis [45]. The 10-year OS rate typically falls within the range of 54% to 89%. Nonetheless, there is a possibility of recurrence in 10% to 25% of cases within the same 10-year period. This recurrence is more common in cases with R1 or R2 resection. In high-risk patients meeting malignancy criteria such as large tumor size, initial dissemination, pleomorphism, necrosis, and a mitosis rate of ≥4 per 10 high-power fields, the risk of metastatic recurrence in the lung, liver, and bone within five years can be as high as 40% [207]. In multivariate analysis, Salas et al. found that radiation therapy (*p* = 0.021), age (*p* = 0.032), and visceral location (*p* = 0.010) are the most important prognostic factors for local recurrence [45,208]. Furthermore, the scoring system for localized and completely resected pleural SFTs was evaluated in a large review, encompassing 113 patients [209]. In 7.1% of the cases, adjuvant therapy (chemotherapy, radiotherapy, or radio-chemotherapy) was performed. OS at five and 10 years was 90.1%, and 85.5%, respectively, although overall relapse was observed in 8% of the patients during the mean follow-up of 13.2 ± 7.3 years [209]. Based on six characteristics (pleural origin, morphology, size, hypercellularity, mitotic index, and presence of necrosis/hemorrhage), Tapias et al. classified tumors into two categories: low risk (69.9%) and high risk (30.1%) of relapse. A significant correlation was demonstrated between the high-risk category and the instance of relapse during follow-up (*p* = 0.004), worse overall survival (*p* = 0.0008), more extensive lung resections (*p* = 0.001), and the use of additional therapies (*p* = 0.009). In the same article, a significant correlation was observed between the number of mitoses > 4–10/HPF and a higher risk of tumor relapse (*p* < 0.001) [209]. In another article, Gold et al. identified the presence of unclear surgical margins (*p* = 0.02), extrathoracic location (*p* = 0.03), and the presence of a malignant component (*p* < 0.01) as prognostic factors of local recurrence [44]. Furthermore, van Houdt et al. confirmed the association between positive surgical margins and a higher relapse rate [124]. Interestingly, Mosquera et al. for the first time described dedifferentiation in eight cases of primary SFTs. This phenomenon was observed among spindled, round epithelioid cells with a loss of CD34 positivity and correlated with a higher risk of relapse [100]. However, Yamada et al. found an association between the occurrence of relapse and hypoglycemia (*p* = 0.001) [210]. In the multivariate analysis, Georgiesh et al. found that late local and distant recurrences are associated with the male sex, while the presence of necrosis and mitotic count ≥ 4 was significantly correlated with the risk of both early and late local and distant relapse. Recurrence was observed in 31% of the patients, with a median time of 63 months [156]. In one of the largest studies of 243 resected extra-pleural and extrameningeal SFTs, a significant correlation was demonstrated among hypercellularity (HR = 1.82, *p* = 0.031), nuclear pleomorphism (HR = 1.62, *p* = 0.015), increased mitotic rate (hazard ratio, HR = 2.85, *p* = 0.002), and recurrence rate [205]. Additionally, TP53 immunohistochemical expression was also found to be associated with relapse (*p* = 0.006) [151]. Ozaniak et al. observed that relapse occurs more often in patients treated for recurrent tumors than newly diagnosed ones [13].

### 11.2. Factors Predicting Metastases

Among SFTs, both clinically benign tumors and rapidly progressing tumors are observed. TNM classification is not used in SFTs [54]. However, there are numerous risk stratification models. Demicco et al. proposed the division of primarily resected SFTs into three categories of risk of poor outcome: low-risk, intermediate-risk, and high-risk tumors based on the presence of high-risk characteristics such as tumors size ≥ 15 cm, age ≥ 55, and mitotic figures ≥ 4/10 (Table 1). In addition, they found that these characteristics were associated with time to metastasis and tumor-related death. The percentages of 110 patients who had not died from a specific disease in five and 10 years were 89 and 73%, respectively [27]. Interestingly, in the next analysis of 79 patients, the modified risk stratification model included necrosis as a predictor of metastasis (*p* = 0.0023). Subsequently, the five-year risk of metastasis in patients in the high- and intermediate-risk classes was at the level of 73% and 10%, respectively [42]. Georgiesh et al. proposed a risk model, termed G-score, including sex, mitotic count, and necrosis, from a cohort with long-term follow-up that also accounted for late recurrences [156,190]. This model seemed to better identify low-risk patients. In a large international collaboration that included 318 patients, the G score and modified Demicco and Salas models were investigated. All models significantly predicted the outcome. The G score was superior in predicting patients with a low risk of relapse, while the Demicco model was superior in identifying high-risk patients [123]. Gold et al. found tumor size >10 cm (*p* < 0.01), positive surgical margins (*p* < 0.01), and malignant component (*p* < 0.01) as a risk factor for metastasis. Interestingly, they proved that tumor size alone does not predict a worse outcome, because great-size tumors with the absence of malignant components were also reported [44]. Furthermore, the histological outlook of SFT may implicate a prognosis. Focal dedifferentiation evaluated on pathomorphological examination was correlated with a higher risk of distant metastases (*p* = 0.001) [211]. Furthermore, dedifferentiation (*p* < 0.0001) also increases the risk of hypoglycemia (*p* < 0.0001) [210]. In another study, the following factors were significantly correlated with metastasis: high mitosis rate (>4/10 HPF) in combination with tumor size >10 cm. Furthermore, the positivity of epithelial membrane antigen (EMA) was also correlated with the risk of dissemination (*p* = 0.03) [124] and immunohistochemical expression [151].

### 11.3. Survival, Prognostic Factors of SFT-Tumor Death, and Overall Survival

In one study on SFTs localized in extremities, the *TERT* promoter mutation significantly correlated with the malignant behavior of the tumors [212]. Different studies also proved this statement [151,160]. Additionally, an analysis showed that the malignant behavior of SFTs is associated with TP53 immunopositivity (*p* = 0.006) and loss of APAF1 immunoreactivity (*p* < 0.001) [151]. Furthermore, another study found that no disease dissemination or death was observed among tumors classified in a low-risk category according to the Demicco criteria [212]. Yamada et al. identified male sex (*p* = 0.0154), larger size (*p* = 0.0455), hypoglycemia (*p* < 0.0001), and dedifferentiation (*p* < 0.0001) to be associated with tumor death. Furthermore, dedifferentiation was a major prognostic factor for overall survival (*p* = 0.0467) [210]. In another study, the following were among the factors significantly correlated with overall survival: high mitosis rate (>4/10 HPF) in combination with tumor size > 10 cm [124] and smooth muscle actin (SMA) positivity was also associated with worse OS (*p* = 0.04). Interestingly, no association between the presence of necrosis and dissemination, relapse, or overall survival was found [124]. In a study of 219 patients, Gholami et al. found tumor size > 8 cm (*p* = 0.05), location in the chest or abdominal/retroperitoneal cavity (*p* = 0.01), and presence of recurrence to be associated with disease-specific death [36]. At the same time, Pasquali et al. observed reduced overall survival in the case of hypercellularity (HR = 1.72, 95%CI 1.03–2.89, *p* = 0.04) and presence (HR = 2.26, 95%CI 1.40–3.66, *p* = 0.001) [205]. In multivariate analysis, Salas et al. found age ≥ 60 years (HR = 1.06; 95% CI = 1.02–1.11; *p* = 0.007) and mitotic activity > 4/10 HPF (HR = 1.03; 95% CI = 01.00–1.07; *p* = 0.060) have a substantial impact on OS [45]. 

## 12. Summary

SFTs are rare mesenchymal tumors with the potential for local recurrence and metastasis. They can occur in various anatomical locations, with the intrathoracic region being the most common. While SFTs are slow-growing, they can exert pressure on adjacent tissues. They remain a diagnostic challenge due to the lack of distinguishing clinical characteristics. SFTs pose diagnostic challenges with a high misdiagnosis rate. Accurate diagnosis requires careful consideration of clinical and histopathological features and exclusion of other malignancies. Diagnosis is confirmed through immunohistochemical staining, which typically shows positive staining for CD34 and negative staining for S-100. Identification of *NAB2-STAT6* gene fusion facilitates the correct diagnosis. Radical surgery with negative resection margins remains the primary treatment approach. Definitive radiotherapy appears to be a promising therapeutic modality. Patients require long-term follow-up due to the possibility of relapse even years after the initial diagnosis, particularly in malignant SFTs. Systemic therapies, such as bevacizumab and tyrosine kinase inhibitors, have shown promise in SFT treatment. Immunotherapy, although not approved, has demonstrated potential in a few cases. While radiotherapy can improve overall survival, targeted therapy and immunotherapy should be explored further. Diagnosis of SFTs often necessitates a combination of imaging studies, histological analysis, and clinical evaluation. Recognizing their characteristic imaging features can aid in accurate diagnosis and management. Several factors have been identified as predictors of local recurrence and metastases. Factors associated with local recurrence include radiation therapy, age, and visceral location. Risk stratification models categorize tumors into low-risk and high-risk groups based on characteristics like pleural origin, morphology, size, hypercellularity, mitotic index, and the presence of necrosis/hemorrhage. Dedifferentiation, unclear surgical margins, extrathoracic location, and a malignant component have also been linked to local recurrence. A global consensus on SFT treatment is needed, along with multidisciplinary approaches to ensure proper management. Since standards for the treatment of advanced and metastatic diseases are not available, further investigations of novel agents are necessary.

## Figures and Tables

**Table 1:** Two models of prediction in SFTs recommended by WHO Classification of Soft Tissue and Bone Tumors, 5th edition.

*RISK FACTOR*	*SCALE*	*MODEL OF METASTASIS PREDICTION*	
		*Demicco EG 2012*	*Demicco EG 2017*
Age (years)	<55 ≥55	0 1	0 1
Mitotic count (mitoses/mm^2^ or mitoses/10 HPFs)	0 (0) 0.5–1.5 (1–3) ≥2 (≥4)	0 1 2	0 1 2
Tumor size (cm)	0–4.9 5–9.9 10–14.9 ≥15	0 1 2 3	0 1 2 3
Necrosis (percentage)	<10% ≥10%	- -	0 1
		POINTS	
RISK	LOW INTERMEDIATE HIGH	0–2 3–4 5–6	0–3 4–5 6–7
